# Lung microbiota: a new hope for treating acute respiratory distress syndrome?

**DOI:** 10.3389/fmicb.2025.1586949

**Published:** 2025-05-30

**Authors:** Yao Tang, Bo Liu, Aijia Ma, Bo Wang, Huaiyu Xiong, Yi Zhou, Jing Yang, Yan Kang

**Affiliations:** ^1^Department of Critical Care Medicine, West China Hospital of Sichuan University, Chengdu, China; ^2^West China Hospital, Institutes for Systems Genetics, Sichuan University, Chengdu, China

**Keywords:** lung microbiota, ARDS, corona virus disease 2019 (COVID-19), prognosis, metabolic products

## Abstract

The lung microbiota, present in healthy individuals, undergoes alterations in different diseases and is closely linked to changes in both systemic and alveolar immunity. These interactions play a crucial role in the onset and progression of numerous diseases. Acute respiratory distress syndrome (ARDS), one of the most severe conditions encountered in intensive care units (ICU), is characterized by high incidence and mortality rates. The pathophysiology of ARDS involves complex mechanisms, including the activation and dysregulation of overlapping pathways related to injury, inflammation, and coagulation, both locally in the lungs and systemically. Notably, alterations in the microbiota may contribute to the pathogenesis of ARDS. Emerging evidence suggests that changes in the lung microbiota are associated with ARDS development, often marked by increased bacterial burden, reduced microbial diversity, and shifts in microbiota composition. In this review, we focus on the regulatory roles of the lung microbiota in ARDS and their therapeutic potential.

## 1 Introduction

Advances in culture-independent microbiology, particularly high-throughput sequencing, have revealed that the lungs harbor complex and diverse microbial communities in healthy individuals (Charlson et al., 2011; Dickson et al., 2016a; Dickson et al., 2017), overturned the long-held belief that lungs are sterile (Laurenzi et al., 1961; Pecora, 1963).

Dysbiosis refers to the disruption of the balance between beneficial and harmful microbes, with a relative increase or decrease in certain microbial populations. Lung microbiota dysbiosis can lead to immune imbalances, resulting in excessive inflammation and subsequent tissue damage (Yang et al., 2020). In chronic respiratory diseases, the diversity of the lung microbiome is a predictor of mortality in chronic obstructive pulmonary disease (COPD) (Leitao Filho et al., 2019), and bacterial burden is linked to disease progression and mortality in idiopathic pulmonary fibrosis (IPF) (Molyneaux et al., 2014; O’Dwyer et al., 2019). Additionally, alterations in community composition predict exacerbations in bronchiectasis (Rogers et al., 2014).

In contrast to chronic respiratory diseases, research on the lung microbiome in acute respiratory failure remains in its early stages. ARDS is a common and serious lung problem that often leads to long-term ventilator use and death in critically ill patients. It is characterized by protein-rich pulmonary edema, hypoxemia, and alveolar inflammation. These pathological features may be driven by changes in the pulmonary microbiome. Conversely, the dysbiosis of lung microbiota may result from alveolar nutrient availability following edema onset (Ruff et al., 2020).

Despite advances in optimizing mechanical ventilation settings and antimicrobial therapies, adjunctive treatment options for ARDS remain limited. The lung microbiome represents an underexplored factor contributing to clinical variation in critical illness. A comprehensive understanding of its mechanistic role in ARDS could facilitate the development of targeted therapeutic interventions.

## 2 Lung microbiota in healthy lung

In most healthy individuals, the lung microbiota harbors a low density but exhibits significant diversity, with various interacting microbial communities. The adult lung microbiota in these individuals is primarily dominated by key genera within the phyla *Firmicutes* and *Bacteroidetes*, forming a “core microbiota” of the lungs (Man et al., 2017). This core microbiota, consisting of genera such as *Streptococcus*, *Prevotella*, *Veillonella*, *Fusobacterium*, and *Haemophilus*, plays a crucial role in maintaining lung homeostasis (Man et al., 2017; Montassier et al., 2023).

Lung immune cells, especially subpopulations of alveolar macrophages and dendritic cells, exert immunoregulatory functions by promoting the generation of regulatory T cells (Soroosh et al., 2013) and secreting anti-inflammatory molecules such as IL-10, transforming growth factor-beta (TGF-β), and prostaglandin E2 (Hussell and Bell, 2014). Resident microorganisms are integral to maintaining pulmonary immune homeostasis through continuous dialog among lung microbiota, immune cells and airway epithelial cells, which are equipped with pattern recognition receptors. However, the composition of the lung microbiota undergoes significant changes in response to pulmonary pathologies, which can disrupt immune homeostasis and influence the progression of these diseases (Lira-Lucio et al., 2020; Sommariva et al., 2020). Emerging evidence suggests that the lung microbiota plays a critical role in shaping the risk and outcomes of pulmonary diseases by modulating both innate and adaptive immune responses (Khatiwada and Subedi, 2020).

*Streptococcus* may indirectly promote the excessive production of CXCL8 (interleukin-8) through pulmonary microbiota dysbiosis, with elevated CXCL8 levels in sputum correlating with increased severity of COPD (Wang et al., 2023).

*Prevotella*, a key part of the airway microbiota, helps activate the innate immune system and protects the respiratory tract. Horn et al. demonstrated that *Prevotella* facilitated the rapid clearance of *Streptococcus pneumoniae* from the lungs and improved the outcomes of *S. pneumoniae* infection by activating neutrophils. Effective neutrophil-mediated clearance requires the recognition of toll-like receptor (TLR) 2, the induction of the pro-inflammatory cytokine tumor necrosis factor-alpha (TNF-α), and the regulation of inflammation by the anti-inflammatory cytokine IL-10 (Horn et al., 2022).

Furthermore, a study showed that specific lung bacteria, including *Prevotella* spp. and *Veillonella* spp., were associated with an increased number of lymphocytes in bronchoalveolar lavage fluid (BALF), TH17 cell-mediated lung inflammation, and a reduced TLR4 response by alveolar macrophages (Segal et al., 2016). Although the lung microbiome likely contributes significantly to mucosal immune homeostasis, its precise role in establishing and maintaining respiratory health remains unclear (Man et al., 2017).

Both chronic lung disease and acute lung injury/ARDS are associated with airway dysbiosis and enrichment of *Pseudomonas*. However, in chronic lung disease, *Haemophilus*, *Streptococcus*, and *Moraxella* are primarily enriched, while in acute lung injury/ARDS, the changes in the lung microbiome are mainly characterized by the enrichment of gut-associated bacteria (i.e., *Bacteroides* spp.) (Yi et al., 2022).

## 3 Microbiome features in patients with traditional ARDS

This section provides an overview of the current understanding of the lung microbiome in traditional ARDS and COVID-19-associated ARDS (Table 1), and a few comparisons between bacterial taxa in traditional ARDS and COVID-19 ARDS (Table 2).

**TABLE 1:** Studies concerning lung microbiota of mechanically ventilated patient in traditional and COVID-19 ARDS.

Study	Publication, y	Region	Type of patients	Number of patients	Date of sample	Type of samples	Analysis	Main results
**ARDS-exp**								
Schmitt et al. (2020)	2020	Germany	Patients with sepsis-induced ARDS following major abdominal surgery	15 ARDS patients	ARDS onset D0, D5, D10	BALF	16S rRNA	Lower α-diversity and increased dominance. A significant reduction in physiological lung flora, particularly anaerobic bacteria such as *Prevotella* spp. and *Veillonella* spp. The α-diversity index negatively correlated with the length of ICU stay and the requirement for mechanical ventilation
Panzer et al. (2018)	2018	United States	Intubated and mechanically ventilated patients with severe blunt trauma	16 ARDS patients	ICU admission; 48 h after admission;	ETA	16S rRNA	The development of ARDS was associated with changes in lung community composition at 48 h, characterized by a relative enrichment of *Enterobacteriaceae* (OTU2119418) and specific taxa, including *Prevotella* (OTU4319899) and *Fusobacterium* (OTU4447248).
**ARDS-p**								
Shen et al. (2022)	2022	China	Hematological patients with pneumonia-related ARDS	22 responders; 28 non-responders	Within the first 24 h of admission	BALF	mNGS	Responders exhibited lower α-diversity and had higher ventilator-free days at day 28. Non-responders with co-infection had a significantly lower survival rate compared to other patients
Hu et al. (2024)	2024	China	Immuno compromised patients with pneumonia-related ARDS	33“type α”, 12 “type β,” 47 “type γ”	Within 48 h of ICU admission	BALF	mNGS	Patients with type γ exhibited lower microbial α-diversity had higher 30-day mortality and fewer ventilator-free days.
Imbert et al. (2024)	2024	France	ARDS patients	Including 11 bacterial CAP-related ARDS	Within 24 h of ICU admission	ETA	16S rRNA	Bacterial α-diversity was significantly lower in the bacterial CAP-related ARDS compared to the COVID-19 ARDS. In bacterial CAP-related ARDS patients, the abundances of *Acinetobacter* sp. and *Actinomyces* sp. were higher compared to influenza-related ARDS patients, while the abundances of *Prevotella* sp. and *Pyramidobacter* sp. were higher compared to COVID-19-related ARDS patients.
Walsh et al. (2017)	2017	United States	Patients for burn and inhalation injury	24 P/F ≤ 300, 24 P/F>300	Within 72 h of hospitalization	BALF	16S rRNA	Enrichment of *Prevotella melaninogenica* was observed in patients with a PaO_2_/FiO_2_ ratio ≤ 300.
**ARDS**								
Zhang et al. (2022)	2022	China	Sepsis-Induced ARDS patients	111 ARDS-p, 45 ARDS-exp	Within 24 h of ARDS diagnosis, D7	BALF	mNGS	ARDS-p was characterized by decreased microbiome diversity, primarily affecting the normal lung microbes. ARDS-exp was characterized by increased microbiome diversity, particularly in conditionally pathogenic bacteria and intestinal microbes. An increase in the background microbiome Bilophila, a genus of intestinal microbes, is likely a risk factor for mortality in ARDS-exp.
Dickson et al. (2016b)	2016	American	ARDS patients	68 ARDS patients; including pneumonia (20, 29.4%), sepsis (18, 26.4%), aspiration (14, 20.6%)	Following enrollment on D3, D7, D14, D21	BALF	16S DNA	The lung microbiota of post-sepsis mice was significantly enriched with numerous bacteria commonly found in the murine gut, including members of the *Bacteroidales* order, *Enterococcus* species, and *Lachnospiraceae* species. A member of the *Bacteroidales* order (*OTU008*) was identified as a key microbial driver of the altered lung communities. Alveolar TNF-α was positively correlated with the relative abundance of *Proteobacteria*.
Kyo et al. (2019)	2019	Japan	Mechanically ventilated patients	40 ARDS patients; including 16 non survivors ARDS patients	within 24 h after intubation	BALF	16S rRNA, NGS	α-diversity was significantly decreased, and the 16S rRNA gene copy numbers tended to increase. Serum IL-6 levels were significantly elevated and positively correlated with the 16S rRNA gene copy number in non-surviving ARDS patients. The copy numbers and relative abundance of Betaproteobacteria were significantly lower in non-survivors. The copy numbers of *Staphylococcus*, *Streptococcus*, and *Enterobacteriaceae* were significantly correlated with serum IL-6 levels in non-survivors.
Dickson et al. (2020)	2020	United States	Critically ill patients	17 ARDS patients; including cardiac arrest (19, 21%), cerebral vascular accident (9, 10%), other (28, 31%)	24 h of ICU admission	BALF	16S rRNA	The bacterial DNA burden in BAL specimens was higher in patients with ARDS. ARDS specimens were more commonly characterized by species of the *Pasteurellaceae* and *Enterobacteriaceae* families. The *Enterobacteriaceae* family was significantly more abundant in ARDS specimens. The gut-associated *Lachnospiraceae* and *Enterobacteriaceae* families were strongly predictive of fewer ventilator-free days, and the *Lachnospiraceae* family was significantly predictive of worse clinical outcomes in critically ill patients.
Montassier et al. (2023)	2023	French	Patients with brain injury requiring invasive mechanical ventilation	52 ARDS	D0, 4 and 7 after ICU admission	BALF	16S rRNA	Microbiota signatures associated with ARDS were characterized by an enrichment of potentially pathogenic respiratory microbes, including *Pseudomonas* and *Staphylococcus*. Based on the presence of *Staphylococcus*, *Ralstonia*, and *Enterococcus*, samples were classified as either “ARDS” or “no ARDS.”
**COVID-19**								
Imbert et al. (2024)	2024	France	ARDS patients	Including 24 COVID-19	Within 24 h of ICU admission	ETA	16S rRNA	In COVID-19-related ARDS patients, the abundances of *Prevotella* sp. and *Streptococcus* sp. were higher compared to both influenza-related and bacterial CAP-related ARDS patients, while the abundance of *Bacteroides* sp. was higher only when compared to influenza-related ARDS patients.
Dickson et al. (2016b)	2020	China	–	8 COVID-19 patients	–	BALF	Meta transcriptome Sequencing	The microbiota in COVID-19 patients was similar to that in CAP patient, characterized by either dominance of pathogens or elevated levels of oral and upper respiratory tract commensal bacteria.
Merenstein et al. (2021)	2021	United States	–	83 critically ill patients with COVID-19	–	ETA	16S rRNA	Low diversity in the ETA microbiome. Frequent outgrowth of potential respiratory pathogens, particularly *Staphylococcus*.
Zacharias et al. (2022)	2021	United States	–	20 critically ill patients with COVID-19	A median of 4 days after hospitalization	Lung	16S rRNA	The lung microbiome exhibited reduced biodiversity. The bacterial community compositions in COVID-19 cases with DAD were significantly different from those in COVID-19 cases with pneumonia.
Gaibani et al. (2021)	2021	Italy	–	24 critically ill patients with COVID-19	–	BALF	16S rRNA	Low diversity in the lower airway microbiome. The lung microbiome was characterized by enrichment of *Pseudomonas alcaligenes*, *Clostridium hiranonis*, *Acinetobacter schindleri*, *Sphingobacterium* spp., *Acinetobacter* spp., and members of the *Enterobacteriaceae* family.
Kullberg et al. (2022)	2022	The Netherlands	–	114 C-ARDS patients	Routine clinical care	BALF	16S rRNA	Patients with increased lung bacterial were less likely to be extubated and exhibited higher mortality rates. Proinflammatory cytokines, such as TNF-α, were associated with elevated microbial burdens.
De Pascale et al. (2024)	2024	Italy	–	70 C-ARDS patients	The first BAL obtained after intubation	BALF	16S rRNA	COVID-19-related ARDS patients with low Crs/low VR exhibited a lung microbiota dominated by *Proteobacteria*. Lung microbiota diversity served as a negative predictor for weaning from IMV and survival. Procalcitonin levels were significantly negatively correlated with *Firmicutes* and positively correlated with *Proteobacteria*.
Sulaiman et al. (2021)	2021	United States		589 C-ARDS patients	–	BALF	16S rRNA, WGS, RNA meta transcriptome	A negative association was observed between bacterial burden in the lungs and survival. Enrichment of the lower airway with the oral commensal *Mycoplasma salivarium* was linked to poor clinical outcomes.

Definition of abbreviations: ARDS-exp, extrapulmonary ARDS; ARDS-p, intrapulmonary ARDS.

**TABLE 2:** Comparisons between bacterial taxa in Traditional ARDS and COVID-19 ARDS.

	Traditional ARDS	COVID-19 ARDS
Phylum	*Betaproteobacteria* ↓ (Kyo et al., 2019)	*Proteobacteria* ↑ (De Pascale et al., 2024)
Class	–	–
Order	–	–
Family	*Pasteurellaceae* and *Enterobacteriaceae* ↑ (Dickson et al., 2020)	–
Genus	*Bilophila* ↑ (Zhang et al., 2022)	*Staphylococcus* ↑ (Merenstein et al., 2021)
	*Pseudomonas* and *Staphylococcus* ↑	–
	*Staphylococcus*, *Ralstonia*, and *Enterococcus*↑ (Montassier et al., 2023)	–
Species	*Prevotella* spp. and *Veillonella* spp. ↓ (Schmitt et al., 2020)	*Prevotella* sp. and *Streptococcus* sp. ↑ (Imbert et al., 2024)
	*Prevotella* sp. and *Pyramidobacter* sp. ↑ (Imbert et al., 2024)	*Pseudomonas alcaligenes*, *Clostridium hiranonis*, *Acinetobacter schindleri*, *Sphingobacterium* spp., *Acinetobacter* spp., and members of the *Enterobacteriaceae* ↑ (Gaibani et al., 2021)
	*Prevotella melaninogenica* ↑ (Walsh et al., 2017)	–
OUT	*Enterobacteriaceae* (OTU2119418), *Prevotella* (OTU4319899) and *Fusobacterium* (OTU4447248) ↑ (Panzer et al., 2018)	–
	*Bacteroidales* order (OTU008) ↑ (Dickson et al., 2016b)	–

### 3.1 Ecological metrics

Several studies have reported that the bacterial DNA burden is higher in patients with ARDS compared to those without ARDS (Dickson et al., 2020; Kyo et al., 2019). Additionally, Kyo et al. found that serum IL-6 levels were significantly elevated in non-survivor ARDS patients and positively correlated with the 16S rRNA copy number (Kyo et al., 2019). Furthermore, Dickson et al. demonstrated that patients with an increased lung bacterial DNA burden had fewer ventilator-free days in critically ill patients (Dickson et al., 2020).

Most studies have shown that alpha diversity is significantly reduced in patients with ARDS (Imbert et al., 2024; Kyo et al., 2019; Schmitt et al., 2020; Zhang et al., 2022). Another study reported that microbial diversity in the lung microbiome of patients with septic ARDS declines progressively over time (Li et al., 2024). However, Zhang et al. found that patients with extrapulmonary infection-induced ARDS exhibited increased microbial diversity (Zhang et al., 2022).

Shen and colleagues observed that responders to early corticosteroid therapy in hematological patients with pneumonia-associated ARDS had lower alpha diversity compared to non-responders. They also found a negative correlation between alpha diversity and levels of inflammatory markers (serum IL-6, IL-8, TNF-α) and CRP in the responders (Shen et al., 2022). In contrast, the microbiota signatures of immunocompromised patients with no obvious inflammatory symptoms and more severe oxygenation failure were characterized by lower alpha diversity and distinct microbial compositions compared to other immunocompromised patients. Hu et al. reported that gut-associated bacteria were more abundant in patients with type α, which is characterized by more active inflammation (Hu et al., 2024).

Interestingly, Walsh et al. found significant differences not in the species dominating the overall community but in the less abundant taxa in patients with burn and inhalation injuries (Walsh et al., 2017). While these taxa did not differ in microbial diversity between patient groups, they exhibited differences in functional diversity, which ultimately had a greater impact on patient outcomes (Dickson et al., 2016a).

Several studies have reported that lower alpha diversity in the lung microbiota is associated with fewer ventilator-free days (De Pascale et al., 2024; Hu et al., 2024; Schmitt et al., 2020). However, Shen and colleagues detected that the responders of corticosteroids had lower alpha diversities and higher ventilator free days in hematological patients with pneumonia-associated ARDS (Shen et al., 2022). One possible explanation is that corticosteroid treatment may alter the lung microbiota composition, though this aspect was not further explored in their study.

### 3.2 Differential flora

In ARDS patients, the abundance of *Prevotella* spp. was found to increase in both direct and indirect lung injury-induced ARDS (Imbert et al., 2024; Panzer et al., 2018; Walsh et al., 2017). However, Schmitt et al. reported a significant reduction in physiological lung flora and mostly anaerobic bacteria, such as *Prevotella* spp. or *Veillonella* spp., in patients with sepsis-induced ARDS (Schmitt et al., 2020).

In patients with bacterial community-acquired pneumonia (CAP)-related ARDS, the levels of *Acinetobacter* spp. and *Actinomyces* spp. were higher compared to those with influenza-related ARDS. When compared to COVID-19-related ARDS patients, only the abundances of *Prevotella* spp. and *Pyramidobacter* spp. were found to be elevated (Imbert et al., 2024).

Additionally, several studies have identified gut-associated *Bacteroides* genus (OTU009) (Dickson et al., 2016b), *Pasteurellaceae* (Dickson et al., 2020), *Enterobacteriaceae* (Dickson et al., 2020; Panzer et al., 2018), and *Fusobacterium* (OTU4319899) (Panzer et al., 2018) were common and abundant in ARDS patients. Furthermore, Dickson et al. found that the relative abundance of *Proteobacteria* was positively correlated with alveolar TNF-α levels, while the typically abundant *Bacteroidetes* phylum showed a negative correlation with alveolar TNF-α concentrations. Furthermore, the gut-associated *Bacteroides* genus (OTU009) was associated with serum TNF-α levels (Dickson et al., 2016b). Four years later, the same team identified the gut-associated *Lachnospiraceae* and *Enterobacteriaceae* families as the taxa most strongly predictive of fewer ventilator-free days (Dickson et al., 2020).

Panzer et al. explored the relationship between microbiota composition at 48 h and inflammation, finding that this relationship was driven by the presence or absence of specific taxa. They suggested that the loss or overgrowth of certain bacteria during the first 48 h in the ICU might contribute to the inflammation observed at this time. Their study revealed that lung bacterial community variation at 0 h was significantly linked to endothelial injury (measured by soluble intercellular adhesion molecule-1), epithelial injury [vascular endothelial growth factor (VEGF)], and inflammation (IL-8) in critically ill blunt trauma patients. At 48 h, community variation was also associated with elevated IL-6 and IL-8 levels. Interestingly, the composition of the microbiota at 0 h was significantly correlated with 48-hour levels of VEGF, receptor for advanced glycation end-products (RAGE), angiopoietin-2 (ANG-2), pentraxin 3 (PENT3), and IL-8 (Panzer et al., 2018).

Montassier et al. identified microbiota signatures associated with ARDS, characterized by an enrichment of potentially pathogenic respiratory microbes, including *Pseudomonas* and *Staphylococcus*, through data harmonization and the pooling of individual patient data. Furthermore, patients with the presence of *Staphylococcus*, *Ralstonia* and *Enterococcus*, had a lower probability of successful extubation (Montassier et al., 2023). Zhang et al. observed that an increase in *Escherichia coli* and *Staphylococcus aureus* in the lungs may serve as risk factors for mortality in ARDS induced by intrapulmonary infections. While no specific pathogens were linked to prognosis, they demonstrated that the increased presence of *Bilophila*, a genus of intestinal bacteria, could be a potential risk factor for death in ARDS caused by extrapulmonary infections. In contrast, *Hydrobacter* might play a protective role in ARDS induced by intrapulmonary infections (Zhang et al., 2022). However, it remains unclear whether *Hydrobacter* is part of the normal lung microbiota or whether it functions independently or synergistically with other microbiomes.

In addition, Kyo et al. discovered that the ratio of the relative abundance of Betaproteobacterial operational taxonomic units (OTUs) to the maximum relative abundance of three other OTUs (*Staphylococcus*, *Streptococcus*, and *Enterobacteriaceae*) was significantly associated with hospital mortality in ARDS patients. They found that the Betaproteobacteria class was strongly negatively correlated with serum IL-6 levels in the non-survivor. Conversely, *Staphylococcus* and *Streptococcus* at the genus level and *Enterobacteriaceae* at the family level were significantly positively correlated with serum IL-6 levels in the non-survivors (Kyo et al., 2019).

## 4 Microbiome features in severe COVID-19 ARDS patients

### 4.1 Ecological metrics

Dysbiosis of the lung microbiome has been observed in COVID-19 patients (Merenstein et al., 2021; Shen et al., 2020). Compared to healthy lung samples, lung microbiota from mechanically ventilated COVID-19 patients exhibited lower diversity (Gaibani et al., 2021; Merenstein et al., 2021; Zacharias et al., 2022). Pascale et al. further reported that patients with reduced lung microbiota diversity had a longer duration of invasive mechanical ventilation (IMV) and higher mortality (De Pascale et al., 2024).

Interestingly, Kullberg et al. found a significant association between bacterial burden and beta diversity in COVID-19-related ARDS among mechanically ventilated patients, suggesting that bacterial overgrowth may drive compositional changes in critically ill lungs. They also discovered that increased bacterial DNA burden correlated with elevated alveolar concentrations of proinflammatory cytokines (TNF-α, IL-6, IL-1). Furthermore, they reported that the overall lung microbiota composition, rather than individual bacterial genera, was linked to successful extubation in COVID-19-related ARDS (Kullberg et al., 2022). Several studies have also indicated that higher bacterial burden is negatively associated with successful extubation (Kullberg et al., 2022) and survival (Sulaiman et al., 2021) in mechanically ventilated patients with COVID-19-related ARDS. These findings are consistent with those observed in traditional ARDS.

### 4.2 Differential flora

Merenstein et al. found that lung microbiome specimens from COVID-19 patients, sampled via endotracheal aspirates (ETA), showed frequent outgrowth of potential respiratory pathogens, particularly *Staphylococcus* (Merenstein et al., 2021). Additionally, Gaibani et al. reported that the lung microbiota of critically ill COVID-19 patients was enriched with *Pseudomonas alcaligenes*, *Clostridium hiranonis*, *Acinetobacter schindleri*, *Sphingobacterium* spp., *Acinetobacter* spp., and *Enterobacteriaceae*. In contrast, COVID-19-negative patients displayed a higher abundance of lung commensal bacteria, such as *Haemophilus influenzae*, *Veillonella dispar*, *Granulicatella* spp., *Porphyromonas* spp., and *Streptococcus* spp. (Gaibani et al., 2021). Compared to influenza-related and bacterial CAP-related ARDS patients, *Prevotella* and *Streptococcus* were more abundant in COVID-19-related ARDS patients, while *Bacteroides* was only more abundant compared to influenza-related ARDS patients (Imbert et al., 2024). However, Shen et al. reported that the microbiota in COVID-19 patients was similar to that in CAP patients, characterized by either the dominance of pathogens or elevated levels of oral and upper respiratory tract commensal bacteria (Shen et al., 2020).

Moreover, Pascale et al. observed that *Firmicutes* dominated the lung microbiota of patients with high respiratory system compliance/predicted body weight (Crs) phenotype, while in COVID-19-related ARDS patients with low Crs/low ventilatory ratio (VR), *Proteobacteria* predominated. They also found a positive correlation between serum procalcitonin and *Proteobacteria*, and a negative correlation with *Firmicutes* (De Pascale et al., 2024). Additionally, Sulaiman et al. discovered that the oral commensal *Mycoplasma salivarum* was enriched in patients who died or required mechanical ventilation for more than 28 days. In contrast, *Prevotella oris* was more abundant in patients with mechanical ventilation duration ≤ 28 days (Sulaiman et al., 2021).

### 4.3 Microbiome features in acute lung injury animal model

This section reviews current understanding of the lung microbiome in ALI/ARDS animal model (Table 3).

**TABLE 3:** Studies concerning lung microbiota of ALI/ARDS in animal model and Therapeutic options.

Study	Publication, y	Inducers of ALI/ARDS model	Therapeutic options; dose; routes of administration	Animals; sex; age	Date of sample	Type of samples	Analysis	Main results
Tian et al. (2022)	2022	LPS; i.p.	–	Sprague-Dawley rats; Male; 8-weeks old	LPS12 h; LPS48 h	Lung	16S rRNA	*Brevibacterium* was found to be correlated with the cytokines TNF–α, IL-10, and IL-6, as well as the hematological percentage of neutrophils. The Wnt, Notch, and chronic myeloid leukemia signaling pathways were associated with IL-1β. The Mitogen-activated protein kinase (MAPK) signaling pathway (specifically yeast) showed a correlation with IL-10. Ascorbate and aldarate metabolism pathways, along with basal transcription factors, were linked to platelet-related indicators.
Poroyko et al. (2015)	2015	LPS; i.p.	–	C57BL/6J mice; –	BAL 72 h after treatment	BALF	16S rRNA	The loss of *Firmicutes*, represented by the *Alicyclobacillaceae* family, and the proliferation of Proteobacteria, represented by the *Brucellaceae* and *Xanthomonadaceae* families.
Mukherjee et al. (2022)	2022	*Plasmodium berghei;* i.p.	–	C57BL/6J; DBA/2 mice; Male	–	Lung	16S rRNA	The parasite in the lung activates the immune system, causing T cells to produce the cytokine IL-10, which disrupts microbial control and promotes MA-ARDS.
Jin et al. (2021)	2022	LPS; –	Vitamin D3; 150 μg/kg; gavages	C57BL/6 mice; Male; 8-10 weeks old	After LPS for 6 h	BALF	16S rDNA, non-targeted metabolomics	The abundance of *Rodentibacter* was positively correlated with the gene expression of IL-1β, IL-6, and TNF-α. Short-term vitamin D3 treatment effectively reduced the abundance of *Rodentibacter*. The abundance of *Rodentibacter* was positively correlated with the gene expression of IL-1β, IL-6, and TNF-α. Short-term vitamin D3 treatment effectively reduced the abundance of Rodentibacter. Short-term vitamin D3 supplementation prevents LPS-induced ALI by inhibiting the production of pro-inflammatory cytokines, including IL-1β, IL-6, and TNF-α. Short-term vitamin D3 significantly reduces the phosphorylation of signal transducer and activator of transcription and suppressor of cytokine signaling 3, while upregulating the phosphorylation of the inhibitor of NF-κB.
Lv et al. (2023)	2023	LPS; i.p.	HUC-MSCs; 0.5 mL PBS containing 1 × 10^6^ HUC-MSCs; i.p.	C57BL/6 mice; Male; 6-8 weeks old	D3 after HUC-MSC	BALF	16S rDNA	HUC-MSCs alleviated pulmonary edema and injury in both the lung and ileum, and reduced mononuclear cell and neutrophil counts, protein concentrations in BALF, and inflammatory cytokine levels in the serum, lung, and ileum of ALI mice. Compared to the LPS group, five metabolites were upregulated, and 11 metabolites were downregulated in the LPS + MSC group.
Mohammed et al. (2020)	2020	SEB; intranasally; i.p.	Δ9-tetrahydro cannabinol; 20 mg/kg; i.p.	C3H/HeJ mice; Female; 8–10 weeks old	D3 after SEB administration	Lung	16S rRNA	THC significantly increased the abundance of the beneficial bacterial species *Ruminococcus gnavus*, while reducing the abundance of the pathogenic microbiota *Akkermansia muciniphila*. THC treatment led to an increase in SCFAs, with propionic acid being identified as a key factor in inhibiting the inflammatory response. THC upregulated several genes, including lysozyme 1 and 2, β-defensin-2, claudin, zonula occludens-1, occludin-1, Mucin2, and Muc5b, while downregulating β-defensin-1
Sultan et al. (2021)	2021	SEB; intranasally	AEA; 40 mg/k; i.p.	C57BL/6 mice; Female; 6–8 weeks old	48 h after SEB	Lung	16S rRNA	AEA treatment attenuates SEB-mediated ARDS by suppressing inflammation and preventing dysbiosis through the induction of AMPs, tight junction proteins, and SCFAs.
Alghetaa et al. (2021)	2023	SEB; intranasally; i.p.	Resveratrol; 100 mg/kg; oral-gavage	C3H/HeJ mice; Female; 6-weeks old	48 h after 2th SEB	Lung	16S rRNA	RES-mediated attenuation of ARDS occurs, at least in part, through the induction of beneficial bacteria, such as *L. reuteri*.
Sapra et al. (2024)	2024	LPS; intranasally/ CLP	*Lactobacillus rhamnosus*; 10^9^CFU/day; orally/SCFA (Butyrate); 200 mM; i.p.	BALB/c and C57BL/6 J mice; Male; 8–10 weeks	24 h after LPS or CLP;	-	-	Pretreatment with LR significantly alleviated pulmonary edema by modulating neutrophil, accompanied by a marked suppression of inflammatory cytokine production in the BALF, lung tissue, and serum. Short-chain fatty acids produced by LR, especially butyrate, targets the phagocytic and neutrophils extracellular traps releasing potential of neutrophils.

### 4.4 Ecological metrics

Poroyko et al. observed a fivefold increase in bacterial load in a mouse model 72 h after intratracheal administration of sterile bacterial wall lipopolysaccharide (LPS) (Poroyko et al., 2015). In addition, Tian et al. found a significant reduction in the volatility of α-diversity 48 hours after LPS was injected intraperitoneally in rats (Tian et al., 2022).

In contrast, Dickson et al. reported that the lungs of post-sepsis mice exhibited increased bacterial diversity 3 days after exposure in a mouse model of lung injury induced by cecal ligation and puncture (CLP) (Dickson et al., 2016b).

### 4.5 Differential flora

In addition, Dickson et al. found that the lung microbiota of post-sepsis mice were significantly enriched with numerous bacteria commonly found in the murine gut, including members of the *Bacteroidales* order, *Enterococcus* species, and *Lachnospiraceae* (sp.). Notably, a member of the *Bacteroidales* order (OTU008) was identified as a key driver of community alterations (Dickson et al., 2016b).

Poroyko et al. observed that the primary microbial response to LPS-induced ALI was characterized by a loss of *Firmicutes*—represented by the family *Alicyclobacillaceae*—and a proliferation of *Proteobacteria*, particularly from the *Brucellaceae* and *Xanthomonadaceae* families (Poroyko et al., 2015).

Previous studies have shown that *Stenotrophomonas* directly contributes to the inflammatory process and impairs respiratory function (Di Bonaventura et al., 2010). Similarly, *Ochrobactrum* has been implicated in lung inflammation (Dawkins et al., 2006; Naik et al., 2013). Additionally, Poroyko et al. detected bacterial substrates suitable for *Ochrobactrum anthropi* and *Stenotrophomonas maltophilia* in BALF, identifying 35 lung metabolites that could serve as substrates. Notably, they found eight metabolites shared by both pathogens, including four carbohydrates, two nucleotide/nucleoside compounds, and two organic acids (citrate and lactate). Furthermore, six metabolites—putrescine, desmosterol, tetracosanoic acid, uric acid, 3-hydroxycholestane, and lactic acid—were significantly elevated in samples from injured lungs (Poroyko et al., 2015).

Additionally, Mukherjee et al. found that the adherence of *Plasmodium*-infected red blood cells to the vascular endothelium induces persistent immune activation, leading to the production of the anti-inflammatory cytokine IL-10 by T cells in the lung. Elevated IL-10 levels promote bacterial expansion in the lung, thereby contributing to the development of malaria-associated acute respiratory distress syndrome (MA-ARDS) (Mukherjee et al., 2022).

Tian et al. found that *Brevibacterium* was positively correlated with serum cytokines TNF-α, IL-10, and IL-6 levels. Additionally, they observed that the functionality of the lung microbiota appeared to be more stable and less variable compared to its composition, with only three differential pathways identified in terms of functional abundance. Changes in the abundance of the ABC transporter pathway may reflect the adaptation of lung bacteria to inflammatory conditions.

The upregulation of bacterial chemotaxis functions in the lung microbiota may result from bacterial adaptation to the edema environment. The reduction in the pentose phosphate pathway could indicate a microbiota with lower antioxidant capacities and diminished DNA repair abilities following lung injury. Regarding lung microbiota functionality, the abundances of four signaling pathways—Wnt (ko04310), Notch (ko04330), chronic myeloid leukemia (ko05220), and MAPK-yeast (ko04011)—were strongly negatively correlated with serum IL-1β and IL-10 levels, suggesting that these pathways may play a role in the interaction between the lung microbiota and host immunity (Tian et al., 2022).

### 4.6 Prospect of therapy

**FIGURE 1:**
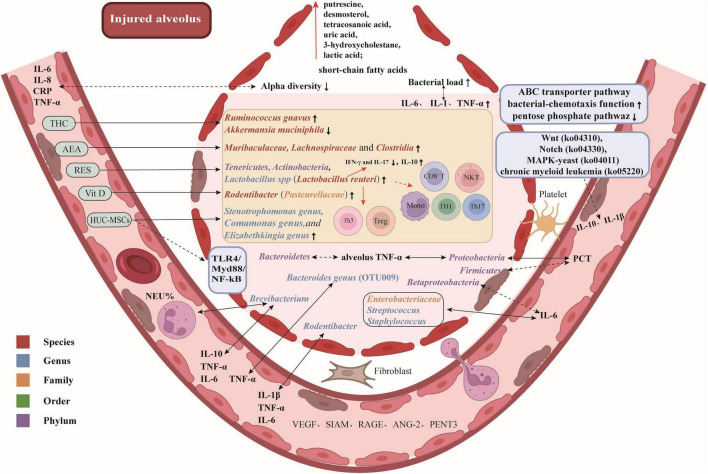
Potential therapeutic targets and promising drugs for attenuating ALI/ARDS by modulating lung microbiota. Gut-associated bacteria, bacteria that are enriched in the gut, such as *Bacteroides* spp.; Responders to early corticosteroid therapy, patients who showed a significant improvement in their PaO_2_/FiO_2_ ratio after receiving corticosteroid treatment. Arterial blood gas measurements were obtained upon ICU admission, prior to the initial administration of corticosteroids, and repeated 24 hours later, following (or prior to) corticosteroid treatment. The difference in PaO_2_/FiO_2_ between these two time points, referred to as ΔPaO_2_/FiO_2_, was calculated. Patients with a ΔPaO_2_/FiO_2_ ≥ 150 mmHg were categorized as responders, while those with a ΔPaO_2_/FiO_2_ < 150 mmHg were classified as non-responders.

Here are some potential therapeutic targets and promising drugs for attenuating ALI/ARDS by modulating the lung microbiota, as illustrated in Figure 1.

### 4.7 The target of potential treatment: microbial community

#### 4.7.1 Anandamide

Endocannabinoids, which are host-derived lipid hormones, possess potent anti-inflammatory properties (Ahmed et al., 2021; Jackson et al., 2014; Osafo et al., 2021; Pandey et al., 2009; Sido et al., 2016). Some studies have suggested that endocannabinoids can modulate the diversity of the gut microbiota (Finn et al., 2021; Minichino et al., 2021). Sultan et al. demonstrated that staphylococcal enterotoxin B (SEB) induces alterations in both the lung and gut microbiota, particularly leading to an overgrowth of pathogenic bacteria such as *Pseudomonas*. However, AEA treatment reversed these changes by promoting the induction of antimicrobial peptides (AMPs) and tight junction proteins. Additionally, AEA treatment enhanced the abundance of beneficial bacteria in the lungs of mice, including *Muribaculaceae*, *Lachnospiraceae*, and *Clostridia*, which are known to produce butyrate (Sultan et al., 2021).

### 4.8 The target of drug intervention: microbial community and immune system

#### 4.8.1 Δ9-tetrahydrocannabinol

Δ9-Tetrahydrocannabinol (THC), a cannabinoid found in *Cannabis sativa* L., is well known for its anti-inflammatory properties (Nagarkatti et al., 2009). Studies have shown that THC and cannabidiol can influence gut microbiota in experimental models of autoimmune diseases (Al-Ghezi et al., 2019; Cluny et al., 2015). Mohammed et al. found that THC administration protected mice from SEB-induced mortality and attenuated ARDS by suppressing lung inflammation and modulating microbial dysbiosis and their metabolites in the lungs. Specifically, they observed that THC increased the abundance of the beneficial bacterial species *Ruminococcus gnavus* in *Firmicutes* phylum, while decreasing the abundance of *Akkermansia muciniphila* (Mohammed et al., 2020). *R. gnavus* is a human gut symbiont known to enhance the expression of glycoproteins (Graziani et al., 2016), which are critical for maintaining lung tissue integrity, preventing vascular leaks, and defending against bacterial pneumonia (Johansson et al., 2011; Roy et al., 2014). In contrast, *A. muciniphila*, commonly found in the intestines of humans and animals, can elevate the expression of pro-inflammatory cytokines (Ganesh et al., 2013) and has a specialized ability to degrade mucin (Derrien et al., 2017; Ganesh et al., 2013).

Additionally, THC was found to significantly increase the levels of short-chain fatty acids (SCFAs) such as propionic acid, butyric acid, and acetic acid in the colon. Notably, propionic acid inhibited the production of pro-inflammatory cytokines while promoting the release of anti-inflammatory cytokines. Furthermore, investigations have shown that *R. gnavus* produces propionate and propanol as end products of metabolism (Crost et al., 2013). These findings suggest that THC may promote the abundance of beneficial bacterial species like *R. gnavus*, enhance the production of SCFAs such as propionic acid, and suppress pro-inflammatory responses, thereby attenuating acute lung injury.

#### 4.8.2 Human umbilical cord mesenchymal cells

A previous study has demonstrated that HUC-MSCs promote the expression of PD-L1 on macrophages and attenuate ALI in mice (Tu et al., 2022). Lv et al. observed that HUC-MSCs can improve pulmonary edema, lung injury, and endothelial barrier function in LPS-induced ALI mice, while also reducing the expression of inflammatory cytokines in both serum and lung tissue. Additionally, HUC-MSC treatment enhanced the integrity of the endothelial barrier in the lungs. More importantly, they found that HUC-MSCs ameliorated ALI by reducing the abundance of pathogenic bacteria, including *Stenotrophomonas*, *Comamonas*, and *Elizabethkingia* genera, in the BALF of mice. Furthermore, *Haemophilus* may play a critical role in the improvement of ALI by HUC-MSCs. Moreover, *Lactobacillus*, *Bacteroides*, and an unidentified *Rikenellaceae* genus were identified as potential biomarkers for evaluating the therapeutic efficacy of HUC-MSCs. The study also revealed that HUC-MSC treatment of ALI mice significantly attenuated the expression of the TLR4/Myd88/NF-κB signaling pathway in lung tissue. Metabolomic analysis further indicated that MSC treatment upregulated 5 metabolites and downregulated 11 metabolites, primarily related to purine metabolism and taste transduction signaling pathways. Notably, metabolites involved in drug metabolism, tyrosine metabolism, autophagy, and endocytosis were significantly associated with the improvement of ALI by HUC-MSC treatment (Lv et al., 2023).

#### 4.8.3 Vitamin D3

Jin et al. observed an increased relative abundance of *Rodentibacter* in the BALF of LPS-treated mice. Short-term vitamin D3 treatment was found to effectively reduce *Rodentibacter* abundance and attenuate ALI. Furthermore, they noted a positive correlation between the abundance of *Rodentibacter* and serum levels of pro-inflammatory cytokines, including IL-1β, IL-6, and TNF-α (Jin et al., 2021).

*Rodentibacter*, primarily found in rodents, belongs to the *Pasteurellaceae* family (Adhikary et al., 2017; Benga et al., 2018), and has been reported as an opportunistic pathogen, or even a primary pathogen, under certain conditions (Benga et al., 2015).

### 4.9 The target of potential treatment: microbial community and immune system

#### 4.9.1 Resveratrol

Resveratrol (RES), a stilbenoid, is well known for its potent anti-inflammatory and antioxidant properties (Chen et al., 2020; Salehi et al., 2018). Studies have shown that RES can significantly alter gut microbiota composition (Alrafas et al., 2019; Li F. et al., 2020), and alleviate SEB-induced ARDS in mice (Alghetaa et al., 2018; Rieder et al., 2012). Alghetaa et al. reported that SEB exposure promotes the growth of pathogenic bacteria, such as those from the *Proteobacteria* phylum and *Propionibacterium acnes* species, in lung microbiota, contributing to ARDS pathogenesis. Their findings indicated that RES treatment significantly enhanced lung microbiota diversity in SEB-exposed mice, increasing the abundance of beneficial bacteria, including the *Tenericutes* and *Actinobacteria* phyla, as well as the *Lactobacillus* genus, particularly *Lactobacillus reuteri*. Colonic microbiota transplantation (CMT) experiments further confirmed that RES-induced enrichment of beneficial bacteria, particularly *L. reuteri*, plays a crucial role in mitigating ARDS. Specifically, *L. reuteri* reduced the number of lung-infiltrating mononuclear cells, cytotoxic CD8+ T cells, NKT cells, Th1 cells, and Th17 cells, while increasing the proportions of regulatory T cells (Tregs) and Th3 cells. Furthermore, *L. reuteri* inhibited the SEB-induced production of pro-inflammatory cytokines such as IFN-γ and IL-17, while promoting the production of the anti-inflammatory cytokine IL-10 (Alghetaa et al., 2021).

*L. reuteri* is a well-studied probiotic bacterium found in various human organs and tissues. It has been shown to produce antimicrobial molecules, which inhibit the colonization of pathogenic microbes (Mu et al., 2018). Studies also suggest that *L. reuteri* can suppress the production of pro-inflammatory cytokines while enhancing the development and function of regulatory T cells (Tregs) (Li L. et al., 2020). Additionally, *L. reuteri* has been linked to increased butyric acid concentrations in the gut, contributing to improved gut health and immune regulation (Li L. et al., 2020; Liu et al., 2017).

#### 4.9.2 *Lactobacillus rhamnosus*

Pretreatment with LR notably improved lung vascular permeability (edema) by modulating neutrophils, while also significantly decreasing the expression of inflammatory cytokines in the BALF, lungs, and serum in both pulmonary and extrapulmonary mouse models. From a mechanistic perspective, LR, through its short-chain fatty acids (with butyrate being the most potent and effective in improving the pathophysiology of both pulmonary and extrapulmonary ARDS), influences neutrophil phagocytosis and their capacity to release extracellular traps. Moreover, butyrate demonstrates enhanced potential in alleviating the pathophysiology of ARDS by reducing neutrophil infiltration into the lungs (Sapra et al., 2024).

## 5 Conclusion and prospects

The lung microbiome undergoes significant alterations in ARDS compared to healthy individuals, with these changes closely linked to disease severity. While the literature partially agrees on the specific microbial changes, certain patterns are consistently observed. ARDS is characterized by an increased bacterial burden and reduced microbial diversity. Patients with lower alpha diversity and higher bacterial loads in their lung microbiota tend to have fewer ventilator-free days and higher mortality rates. Physiological lung flora, such as *Firmicutes*, is notably diminished, while respiratory pathogens, including *Staphylococcus*, *Streptococcus*, *Enterobacteriaceae*, *Pseudomonas*, and *Proteobacteria*, proliferate in ARDS patients. Furthermore, the lung microbiome is closely associated with immune and inflammatory responses in ARDS. However, the impact of different initial infection sites on lung microbiome diversity and composition remains unclear, and study heterogeneity may significantly influence findings. We summarized that these studies involved populations with different causes of ARDS, varying in sample sizes, countries and regions, all of which may contribute to the inconsistency of the findings. Given that lower airway microbiota composition can vary depending on the underlying cause of ARDS, future research and experimental models should account for the etiology when investigating the role of the lung microbiome in ARDS pathophysiology.
